# Corneal Confocal Microscopy in the Diagnosis of Small Fiber Neuropathy: Faster, Easier, and More Efficient Than Skin Biopsy?

**DOI:** 10.3390/pathophysiology29010001

**Published:** 2021-12-26

**Authors:** Mariia V. Lukashenko, Natalia Y. Gavrilova, Anna V. Bregovskaya, Lidiia A. Soprun, Leonid P. Churilov, Ioannis N. Petropoulos, Rayaz A Malik, Yehuda Shoenfeld

**Affiliations:** 1 Laboratory Mosaic Autoimmunity, St. Petersburg State University, 199304 St. Petersburg, Russia; pushisti.legolas@mail.ru (M.V.L.); lidas7@yandex.ru (L.A.S.); elpach@mail.ru (L.P.C.); yehuda.shoenfeld@sheba.health.gov.il (Y.S.); 2 Department of Phthisiopulmonology, St. Petersburg Scientific Research Institute of Phthisiopulmonology, 199304 St. Petersburg, Russia; 3 Department of endocrinology, Almazov National Medical Research Centre, 199304 St. Petersburg, Russia; anna_breg@inbox.ru; 4 Department of Medicine, Research Division, Weill Cornell Medicine-Qatar, Doha 24144, Qatar; inp2002@qatar-med.cornell.edu (I.N.P.); ram2045@qatar-med.cornell.edu (R.A.M.); 5 Zabludowicz Center for Autoimmune Diseases, Sheba Medical Center, Tel HaShomer 52621, Israel; 6 Sackler Faculty of Medicine, Tel-Aviv University, Tel Aviv 69997801, Israel; 7 President, Ariel University, Ariel 4077625, Israel

**Keywords:** small fiber neuropathy (SFN), confocal microscopy (CM), cornea, skin biopsy, autoimmune neuropathies, autoimmunity, sarcoidosis, Sjogren’s syndrome

## Abstract

Chronic pain may affect 30–50% of the world’s population and an important cause is small fiber neuropathy (SFN). Recent research suggests that autoimmune diseases may be one of the most common causes of small nerve fiber damage. There is low awareness of SFN among patients and clinicians and it is difficult to diagnose as routine electrophysiological methods only detect large fiber abnormalities, and specialized small fiber tests, like skin biopsy and quantitative sensory testing, are not routinely available. Corneal confocal microscopy (CCM) is a rapid, non-invasive, reproducible method for quantifying small nerve fiber degeneration and regeneration, and could be an important tool for diagnosing SFN. This review considers the advantages and disadvantages of CCM and highlights the evolution of this technique from a research tool to a diagnostic test for small fiber damage, which can be a valuable contribution to the study and management of autoimmune disease.

## 1. Introduction

Small fiber neuropathy (SFN) can be described as a dysfunction of the nerve fibers of the smallest diameter (A delta and C-types) that make up 70–90% of the entire peripheral nervous system. SFN arises as a consequence of small nerve fiber dysfunction, or damage due to metabolic, toxic, inflammatory, or autoimmune causes [1,2]. There are a wide variety of clinical manifestations in SFN, the most common being pain, dysesthesia, and dysautonomia. Sensory complaints occur in either a classic manifestation of polyneuropathy (stocking/gloves pattern, length-dependent type), or in a non-length-dependent pattern in which more proximal parts of the patient’s body are affected [3]. The management of patients with SFN is challenging, especially with a lack of clear diagnostic criteria, limited understanding of the underlying pathophysiology, and relatively ineffective treatments [4].

The epidemiology of this entity is difficult to define due to the complexity of the diagnosis and the low awareness of the disease among both patients and healthcare providers. A US study showed that the incidence of SFN was 1.3/100,000 per year with a prevalence of 13.3 per 100,000 [5]. However, an earlier study from the Netherlands reported the minimum incidence as 11.73 cases/100,000 and a minimum prevalence of—52.95 cases/100,000 [6]. Possible explanations for such varying rates may be the different etiological factors in different populations, genotype variability and different methods to establish the diagnosis of SFN.

SFN develops due to the involvement of small fibers, and yet many patients remain undiagnosed, and their complaints are considered to be psychogenic due to the absence of large fiber abnormalities on electroneuromyography. The diagnosis of SFN requires an assessment of neuropathic symptoms and neurological signs with a specialized evaluation of small fibers, an assessment of severity, and a visual analog scale. Diagnostic criteria for idiopathic SFN have been established recently and require at least one SF symptom and one SF sign with abnormal intraepidermal small fiber density (IENFD), normal sensory nerve conduction studies, and the absence of large nerve fiber symptoms and signs [7]. Validated SFN questionnaires include “SFN symptoms inventory questionnaire” (SFN-SIQ), “SFN-Rasch-built overall disability scale” (SFN-RODS), “Douleur Neuropathique 4 Questions” (DN4), and “Composite Autonomic Symptom Score” (COMPASS-31) [8].

The punch skin biopsy technique with immunohistochemical staining of the small nerve fibers has significantly improved the diagnostic yield for SFN [8]. However, along with the relatively high cost, this technique cannot be repeated on the same area of skin, it is time consuming, requires a histological laboratory, and can lead to infection and bleeding [9], especially in patients with type 2 diabetes or impaired glucose tolerance, which comprises up to 56% of all patients with SFN [10].

Corneal confocal microscopy (CCM) is an alternative, non-invasive procedure for the evaluation of small fibers in the cornea [11]. Automated analysis and standardized evaluation criteria make the method appropriate for clinical application [12]. It is a rapid, non-invasive ophthalmic technique which can be deployed in pediatric and adult patients with chronic diseases. It allows repeated analysis of the same part of the cornea, which is important for longitudinal assessment to identify disease progression and to assess the effectiveness of therapy. It can also evaluate the severity of underlying autoimmune and inflammatory processes by visualizing dendritic cell morphology and density [13,14].

It should be noted that, in patients with metabolic, toxic, or drug-induced SFN, damage to both small and large nerve fibers is often described and neuropathy usually is “mixed”. These patients have changes both in electroneuromyography and in the above-mentioned methods for diagnosing small fiber neuropathy. At the same time, in autoimmune cases with SFN development, neuropathy is most often affects small fibers and very rarely involves nerves of a larger diameter. Damage to the thin A delta and C-fibers is probably cytokine-mediated via TNF alpha, IL-2, IL-6, and IL-8, but it has not been fully studied [12]. The application of sensitive diagnostic methods to identify SFN is key to the early diagnosis of small fiber damage due to metabolic diseases such as identification of small fiber damage in other neurologic diseases.

## 2. Etiology and Manifestations of Small Fiber Neuropathy

Five main etiological processes underlie SFN (Table 1) [15,16], although approximately 40% of cases remain idiopathic [15,16,17]. Previously, it was considered that most cases of SFN were associated with diabetes mellitus type 1 and 2. However, there is growing evidence that autoimmune conditions may have a greater impact on SFN development: first, because small fiber polyneuropathy was described in various autoimmune diseases, such as Sjogren’s syndrome, celiac disease, and systemic lupus erythematosus [18]; second, in some types of idiopathic SFN, antibodies to the proteins of the nervous tissue were detected, e.g., antibodies to potassium channels or to nicotinic receptors; third, when performing a skin biopsy, an increase in pro-inflammatory cytokines was described in a length-dependent manner (e.g., cytokine titers were higher in more distal parts of the body compared to the more proximal ones); and fourth, patients with this kind of neuropathy described beneficial effects with the treatment of intravenous immunoglobulins or inhibition of TNF-alpha [12].

SFN is characterized by chronic burning pain in the distal limbs, which may also affect the proximal back, chest, and face, and should last more than 6 months [19,20]. Sensory manifestations include hyperalgesia, paresthesia, dysesthesia (itching, tingling, and stitching sensations), and allodynia (a pain response to a non-painful stimulus) [17,21,22]. Dysautonomia of the exocrine glands (sweat, salivary, and lacrimal), smooth muscle in the gastrointestinal tract, urinary, and cardiovascular systems [23,24], and altered hyperaemic and pressure-induced vasodilatation in the skin may contribute to foot ulceration and poor wound healing [25].

## 3. Corneal Confocal Microscopy: Discovery and Method

Confocal microscopy (CM) was discovered more than 75 years ago. The first confocal system was invented in 1943 and, in 1951, Hiroto Naora published the first article on CM in the journal of spectrophotometry [26]. The first commercial corneal confocal microscope (CCM) was a tandem-scanning microscope and was developed in the 1960s by the Czechoslovak scientist Moymir Petrash. Evolution of the scanning systems [24] in combination with laser technology [26] enabled the enhancement of image quality. Currently, there are three different models of CCM: laser scanning CCM, slit-scanning CCM, and tandem-scanning CCM, differing in light emission, resolution, and magnification [27]. The Heidelberg corneal module (HRT-RCM) is the most widely used CCM and has been used to quantify small nerve fibers in a range of peripheral and central neurodegenerative diseases [28].

**Figure 1:**
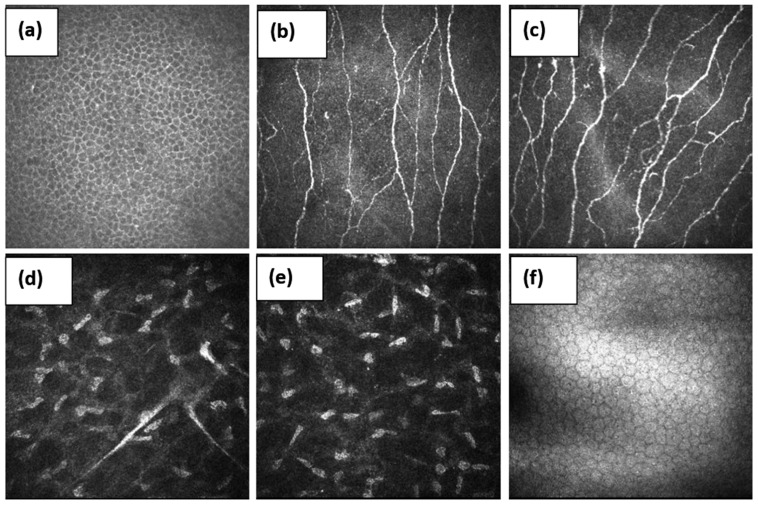
Layer-by-layer image of the healthy cornea. (**a**)—superficial epithelial cells, (**b**,**c**)—sub-basal nerve plexuses, (**d**,**e**)—stroma, and (**f**)—endothelium.

CCM generates high-resolution images of the cells and sub-basal nerve plexus in the cornea [27]. Real-time, in vivo images are obtained without distortion unless there are pressure lines or fixation artifacts since, unlike skin biopsies, the material does not need to be fixed (Figure 1).

The imaging procedure is relatively simple and the technique can be learnt by investigators without an ophthalmic background with 2–3 days of intensive training. The CCM procedure includes: (1) preparation of the tip of the camera lens using gel (Viscotears) and a sterile nozzle (TomoCap); (2) setting the focal plane with the lens focused on the front cover of the TomoCap; (3) application of the local anaesthetic and lubricant gel on the front of the eye; (4) moving the TomoCap 5–10 mm away from the eye with the optical axis of the microscope chamber passing through the center of the anterior pole of the cornea to capture a central image; (5) moving the camera forward until the TomoCap touches the cornea and creates a thin gel bridge; and (6) the HRT III laser camera captures optimal images of the corneal structures using the section mode [29].

Several image analysis software methods have been used to assess the corneal sub-basal nerve plexus. Corneal nerve fiber density (CNFD) (fibers/mm^2^), branch density (CNBD) (branches/mm^2^), and fiber length (CNFL) (total fiber length mm/mm^2^) are most commonly quantified using manual and automated image analysis software [30,31] and a normative range has been established, taking into account age and gender in a large, healthy control cohort [32]. Recently, artificial intelligence (AI) -based deep learning algorithms have been applied to augment corneal nerve analysis and the classification of patients with and without diabetic neuropathy [33,34].

Corneal confocal microscopy is a simple, reproducible, and precise method, but the limitations are also presented. First of all, special equipment and trained personnel are required. However, there is no need for a histological laboratory, which is necessary for a skin biopsy [35]. Patient images are stored in a digital database and can be interpreted both automatically and semi-automatically. The semi-automatic method, in general, is more accurate; however, due to the presence of the human factor, it can also carry errors and limitations. An important feature in the context of autoimmune neuropathies is the need to assess the patient’s local immune status. Due to the fact that this method is non-invasive, the tissue concentrations of cytokines and other immunological factors cannot be evaluated; however, it is possible to assess the number and morphology of dendritic cells, which is an important parameter to investigate in autoimmune conditions.

## 4. Corneal Confocal Microscopy in Small Fiber Neuropathy

**Figure 2:**
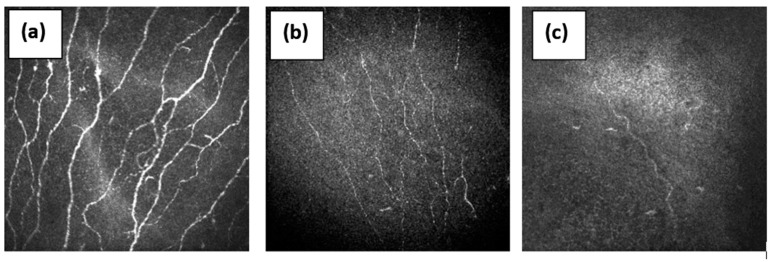
The sub-basal nerve plexus in (**a**)—healthy volunteer with normal nerve fibers, (**b**)—patient with diabetic neuropathy with reduced nerve fibers, and (**c**)—patient with diabetes and Charcot neuro-osteoarthropathy with a severe loss of nerve fibers.

CCM is gaining acceptance in the diagnosis of small fiber neuropathy (Figure 2) [36]. Earlier studies of patients with idiopathic SFN demonstrated decreased corneal nerve fiber density and increased tortuosity [29,36] with increased dendritic cells, indicating systemic inflammation [15]. In a recent deep phenotyping study of patients with idiopathic SFN, whilst abnormalities occurred in the distal intraepidermal nerve fiber density (IENFD) (60/86, 70%) and neurological examination (53/86, 62%) most frequently reflected small fiber disease, adding CCM and/or pain-related evoked potentials (PREP) further increased the identification of patients with small fiber impairment to 47/55 (85%), whilst quantitative sensory testing (QST), quantitative sudomotor reflex testing (QSART), and proximal IENFD were of lower impact [15,37].

Several studies have shown the utility of CCM in both early diagnosis and management of patients with diabetes. Azmi et al. undertook an oral glucose tolerance test, evaluation of neuropathic symptoms, skin biopsy to evaluate intraepidermal nerve fibers density (IENFD), and mean dendrite length (MDL) and CCM to quantify corneal nerve morphology in 30 subjects with impaired glucose tolerance (IGT) and 17 controls at baseline and annually over 3 years. Ten participants with IGT who developed type 2 diabetes had a significantly lower CNFD, CNBD, and CNFL, whilst there was no difference in IENFD or MDL compared to controls. They also showed a further decrease in CNFL, IENFD, and MDL over 3 years. The remaining fifteen subjects with impaired glucose tolerance and five subjects who returned to normal glucose tolerance did not differ significantly in CNFD, CNBD, CNFL, or IENFD [38]. Differences were revealed between patients with painful and painless diabetic neuropathy with a lower corneal nerve fiber density (*p* = 0.005), branch density (*p* = 0.03), length (*p* = 0.01), cold sensation threshold (*p* < 0.0001), and higher warm sensation threshold (*p* = 0.004) [39,40]. For the diagnosis of diabetic neuropathy, CCM, compared to a skin biopsy, has a slightly higher sensitivity (0.77 versus 0.61) and comparable specificity (0.79 versus 0.80) [14]. CCM has been shown to have good diagnostic utility for diabetic neuropathy [41] and can predict the development of diabetic neuropathy [42,43].

Corneal nerve loss also occurs in patients with painful sarcoid neuropathy [44], painful human immunodeficiency virus (HIV) neuropathy [45], chemotherapy-induced peripheral neuropathy, and fibromyalgia [46].

There may be a difference in CCM results in patients with autoimmune and metabolic causes of SFN [47]. Brines M. et al. showed the possibility of differential diagnosis of the SFN of metabolic (type 2 diabetes mellitus) and autoimmune (sarcoidosis) origin with CM, where correlation between CCM variables was higher in metabolic disease than it was for the patients with sarcoidosis [2]. The possibility for utilizing CCM in the differential diagnosis of SFN is promising, though understudied.

Considering that SFN may have both length-dependent (classical polyneuropathy) and non-length-dependent (“patchy”) patterns, it is important to perform studies comparing CCM with other diagnostic techniques, especially skin biopsy and QST in different neuropathies. In patients with sarcoidosis CCM was a more sensitive method which detected SFN in 45% of patients, while a skin biopsy only identified SFN in 28% of patients [48]. Previously, the same authors compared CCM and quantitative sensory testing (QST) results in fibromyalgia (FM) patients. Small fiber pathology was detected in the cornea of half of the patients with FM and four further subtypes were identified based on abnormalities in CCM and central sensitization [49].

## 5. Conclusions

Corneal confocal microscopy has a robust evidence base for the assessment of SFN, especially in diabetic neuropathy [50]. There is an increasing body of data for the utility of CCM in the assessment of other peripheral and central neurodegenerative diseases with underlying autoimmune etiologies. It is a non-invasive, reiterative technique with good reproducibility using manual and automated quantification techniques, enabling rapid diagnosis. Further studies utilizing CCM are needed to expand the utility of this technique in the diagnosis of patients with small fiber neuropathy.

## Figures and Tables

**Table 1:** **The** most common causes of small fiber neuropathy [1,3,4,5,6,7].

Causes	Diseases
Metabolic	Diabetes mellitus Impaired glucose tolerance Vitamin B12 and B6 deficiency Dyslipidemia Chronic hypothyroidism Chronic kidney disease
Infectious	HIV Hepatitis C Lyme Disease Leprosy
Toxic	Alcohol Antibiotics (metronidazole, linezolid) Statins
Autoimmune/inflammatory	ASIA-syndrome Sjogren’s syndrome Sarcoidosis Celiac disease Rheumatoid arthritis Systemic lupus erythematosus Amyloidosis
Hereditary	Fabry disease Tangier’s disease Friedreich Ataxia Familial amyloidosis (mutation of transthyretin) Sodium channels mutations Nav1.8, 1.7
